# HPV42 – a human papillomavirus classified as a low‐risk type with oncogenic potential

**DOI:** 10.1111/ddg.15981

**Published:** 2025-10-06

**Authors:** Sven Niklas Burmann, Ulrike Wieland, Steffi Silling, Frank Oellig, David Chromy, Julia Hyun, Thilo Gambichler, Alexander Kreuter

**Affiliations:** ^1^ Department of Dermatology Venereology and Allergology Helios St. Elisabeth Hospital Oberhausen University Witten/Herdecke Oberhausen Germany; ^2^ Institute of Virology National Reference Center for Papilloma‐ and Polyomaviruses University of Cologne Medical Faculty and University Hospital Cologne Cologne Germany; ^3^ Institute of Pathology Mülheim an der Ruhr Germany; ^4^ University Department of Dermatology Medical University of Vienna Vienna Austria; ^5^ Department of Dermatology Venereology and Allergology Helios St. Johannes Klinik Duisburg Duisburg Germany; ^6^ Department of Dermatology Christian Hospital Unna Unna Germany; ^7^ Department of Dermatology Klinikum Dortmund gGmbH University of Witten/Herdecke Dortmund Germany

**Keywords:** Anogenital warts, condylomata acuminata, digital papillary adenocarcinoma, human papillomavirus, HPV42, squamous cell carcinoma

## Abstract

Human papillomavirus (HPV) type 42 is a rare HPV type currently classified as “low‐risk” and belongs to the alpha genus. It has primarily been detected in benign vulvar papillomas and is predominantly associated with benign lesions such as anogenital warts. However, in very rare cases, HPV42 has also been identified in invasive genital tumors, particularly cervical carcinomas. In the case series presented here, HPV42 was detected in anogenital warts, a digital squamous cell carcinoma in situ, and a lymphogenously metastasized digital papillary adenocarcinoma (DPA). In the latter tumor entity, HPV42 was recently identified as the potential causative agent. Depending on the infected cell population, HPV42 can give rise to both benign lesions (anogenital warts and the rare cervicovaginal “seborrheic keratosis‐like” lesion) and malignant lesions (anogenital and digital carcinomas, as well as the highly aggressive DPA). HPV42 can therefore be considered as potentially carcinogenic. With the currently available nonavalent HPV vaccine over 90% of HPV‐related tumors and anogenital warts can be prevented, but not HPV42‐induced lesions. These tumors will therefore not be suppressed by the current vaccination programs, even not in countries with high vaccination rates.

## INTRODUCTION

Infections with human papillomaviruses (HPV) are among the most common infections in humans. Currently, more than 220 HPV types have been fully classified and can be divided into different genera (alpha‐, beta‐, gamma‐, mu‐, and nu).^1^, ^2^ Approximately 40 HPV types belong to the alpha genus and primarily infect the anogenital region. They can be further divided into high‐risk (HR) and low‐risk (LR) HPV types, depending on their oncogenic potential. HR‐HPVs (e.g., HPV16 and HPV18) cause anogenital carcinomas and their precursors as well as oropharyngeal carcinomas, whereas LR‐HPVs (e.g., HPV6, HPV11) induce benign lesions such as anogenital warts (AGW). In rare cases, LR‐HPVs can also cause intraepithelial neoplasias and invasive carcinomas, as seen in Buschke‐Löwenstein tumors.^3^, ^4^

HPV42 belongs to the papillomavirus genus alpha and has a close phylogenetic relationship with HPV32, the causative agent of focal epithelial hyperplasia. HPV42 is currently classified as an LR‐HPV type and was firstly detected in benign vulvar papillomas in 1982.^5^ In larger studies, HPV42 was found in 1%–2% of examined AGWs.^6^, ^7^ In a global study including over 13.000 HPV‐induced anogenital carcinomas, HPV42 was detected in only four invasive tumors.^8^ Recently, HPV42 was identified in tumor cells of digital papillary adenocarcinomas (DPA), a very rare aggressive tumor predominantly located on fingers and toes.^9^ We herein report clinically and histopathologically different lesions in three patients in whom HPV42 was detected as a monoinfection.

## CASE SERIES

### Patient 1

A 29‐year‐old man with HIV infection who has sex with men presented to our clinic due to recurrent perianal AGWs. HIV infection (stage A2 according to CDC/WHO) was diagnosed in 2018. Since then, he has been on antiretroviral therapy (ART) consisting of raltegravir and emtricitabine/tenofovir alafenamide. At the time of initial presentation, the CD4 T‐cell count was 800/µl, and HIV‐RNA was undetectable in the blood plasma (< 40 copies/ml). Clinically, multiple anogenital warts (AGWs) up to 1 cm in diameter were observed. According to current AWMF guidelines, screening for anal dysplasias was offered, and a high‐resolution anoscopy (HRA) was performed, which revealed no additional anal warts or dysplasias intraanally.^10^ Histopathologically, the perianally excised flat lesions showed acanthotically thickened squamous epithelium with mild basal cell hyperplasia and dense inflammatory cell infiltrates. Immunohistochemical staining for Ki‐67 revealed single suprabasal nuclei, whereas staining for p16INK4a was entirely negative (Figure 1a–d). HPV typing from one lesion using alpha‐HPV group‐specific A6/A8 PCR, which covers 43 HPV types, demonstrated a monoinfection with HPV42.^11^ The patient continues annual HRA follow‐up and has been recurrence‐free for 3 years.

**FIGURE 1:**
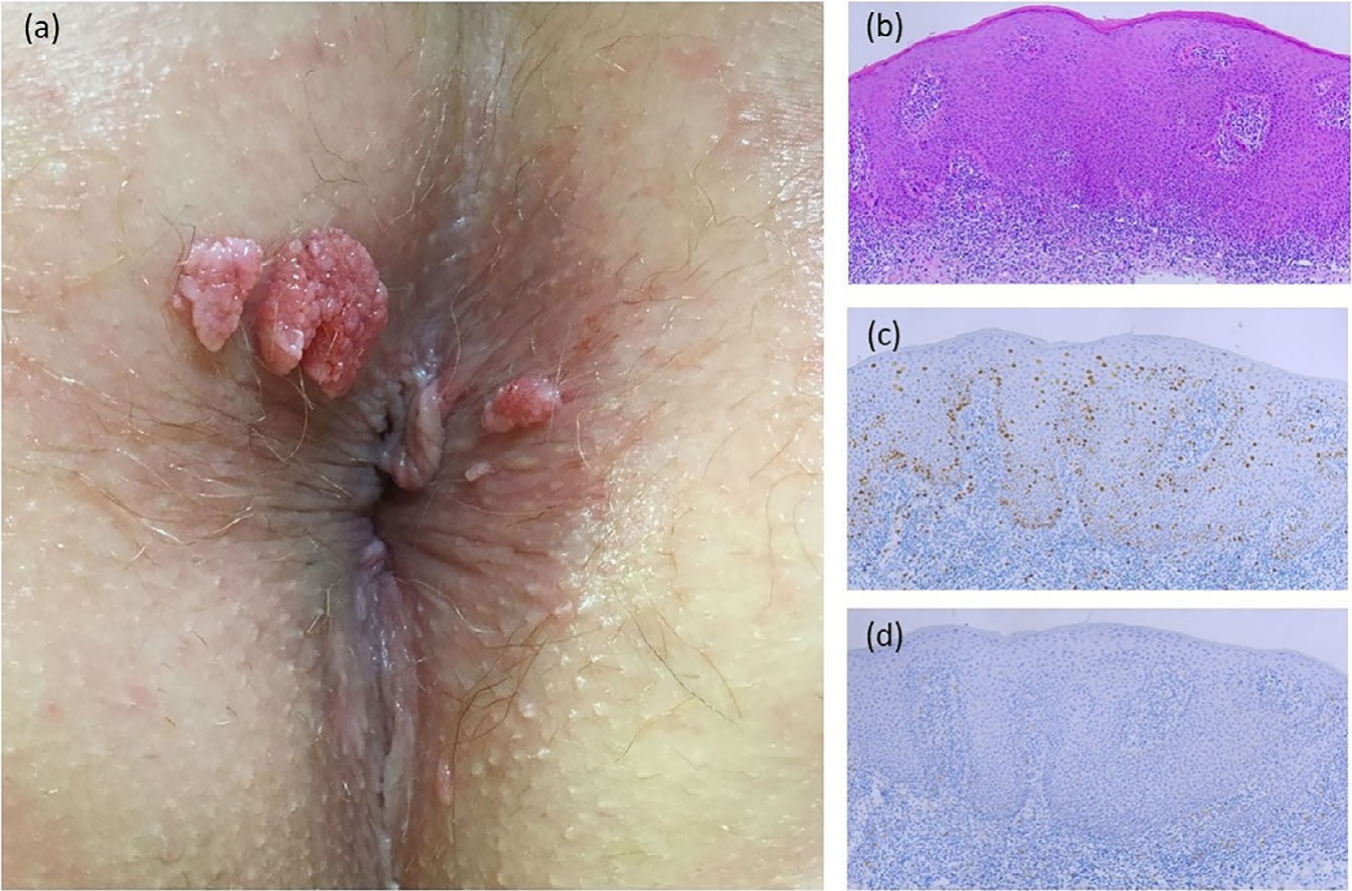
29‐year‐old patient with HIV and HPV42‐positive condylomata acuminata. (a) Clinical presentation at first visit showing perianal condylomata acuminata. (b) Histopathology reveals acanthotic thickening of dysplasia‐free squamous epithelium with mild basal cell hyperplasia and moderately dense inflammatory cell infiltrates (hematoxylin and eosin stain, original magnification × 100). (c) Immunohistochemical staining for Ki‐67 highlights single suprabasal nuclei (Ki‐67, × 100). (d) Immunohistochemistry for p16INK4a expression is negative throughout the epithelium (p16INK4a, × 100).

### Patient 2

A 65‐year‐old male patient presented with a slowly growing, approximately two‐year‐old blackish pigmented, sharply demarcated verrucous plaque on the fourth digit of the right hand. Apart from nicotine abuse, no relevant comorbidities were present. The patient denied risk factors such as increased lifetime ultraviolet radiation exposure, immunosuppression, or intake of photosensitizing substances like hydrochlorothiazide. Histopathological examination of the surgically excised lesion revealed marked epidermal thickening with atypical keratinocytes and dyskeratosis without dermal infiltration. Immunohistochemical staining for Ki‐67 showed positively stained nuclei extending to the upper epidermal layers, along with a band‐like expression of p16INK4a. Based on these findings, a diagnosis of verrucous squamous cell carcinoma (SCC) in situ was made (Figure 2a–d). After histologically controlled complete excision, HPV analysis via A6/A8 PCR^11^ confirmed HPV42 monoinfection. At the last clinical follow‐up 2 years after primary excision of the digital SCC in situ, the patient remained tumor‐free.

**FIGURE 2:**
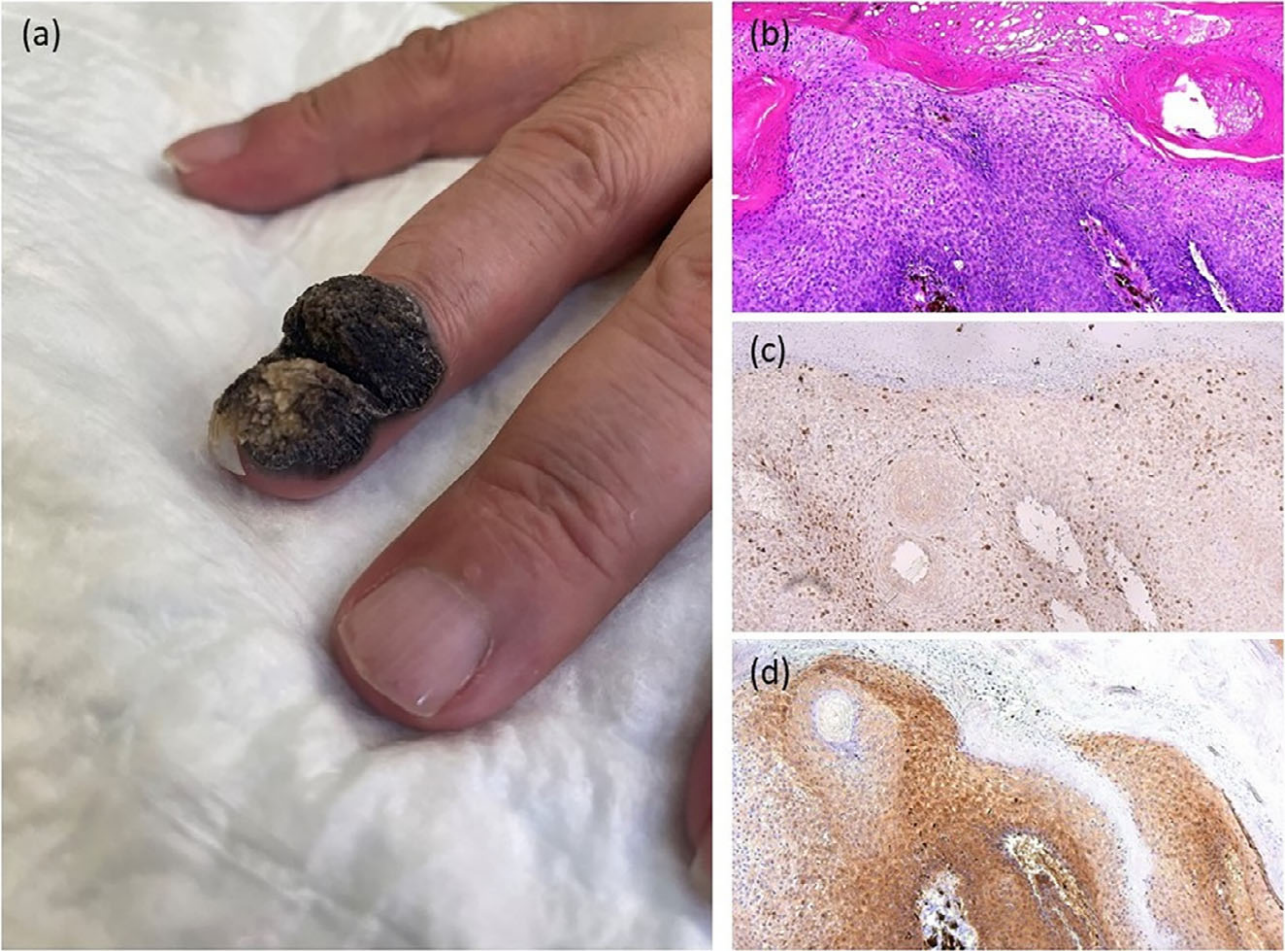
65‐year‐old patient with HPV42‐positive peri‐ and subungual squamous cell carcinoma in situ of the fourth digit of the right hand. (a) Clinical finding at first presentation. (b) Histopathological examination shows epidermal thickening with atypical keratinocytes and dyskeratosis throughout the epithelium, without dermal infiltration (hematoxylin and eosin stain, original magnification × 100). (c) Ki‐67 immunostaining reveals nuclear positivity extending into the upper layers of the epidermis (Ki‐67, × 100). (d) A band‐like (“block”) expression of p16INK4a is observed (p16INK4a, × 100).

### Patient 3

A 52‐year‐old male patient presented with a repeatedly bleeding, progressively enlarging plaque on the distal phalanx of the left middle finger lasting several months. No B‐symptoms were present at the time of initial presentation. No relevant prior illnesses, such as immunosuppression, were reported. Palpation revealed an enlarged lymph node in the left axilla. Sonographically, a highly suspicious, enlarged metastatic lymph node was identified. Computed tomography of the abdomen and thorax as well as MRI of the skull revealed no additional pathological findings. The tumor excision specimen from the finger showed glandularly organized tumor cells with predominantly basophilic cell populations and fibrotic stroma. Immunohistochemical staining for Ki‐67 indicated a markedly increased proliferation rate of more than 80% of the tumor cells, and continuous nuclear and cytoplasmic expression of p16INK4a was present (Figure 3a–e). HPV typing via A6/A8 PCR^11^ detected HPV42 monoinfection in both the primary tumor and the lymph node metastasis. Based on the overall findings, a diagnosis of lymphogenously metastasized DPA was made. Following presentation at the interdisciplinary tumor board, adjuvant radiotherapy of the left axilla with a total dose of 45 Gy was administered. The patient has remained tumor‐free for 4 years since.

**FIGURE 3:**
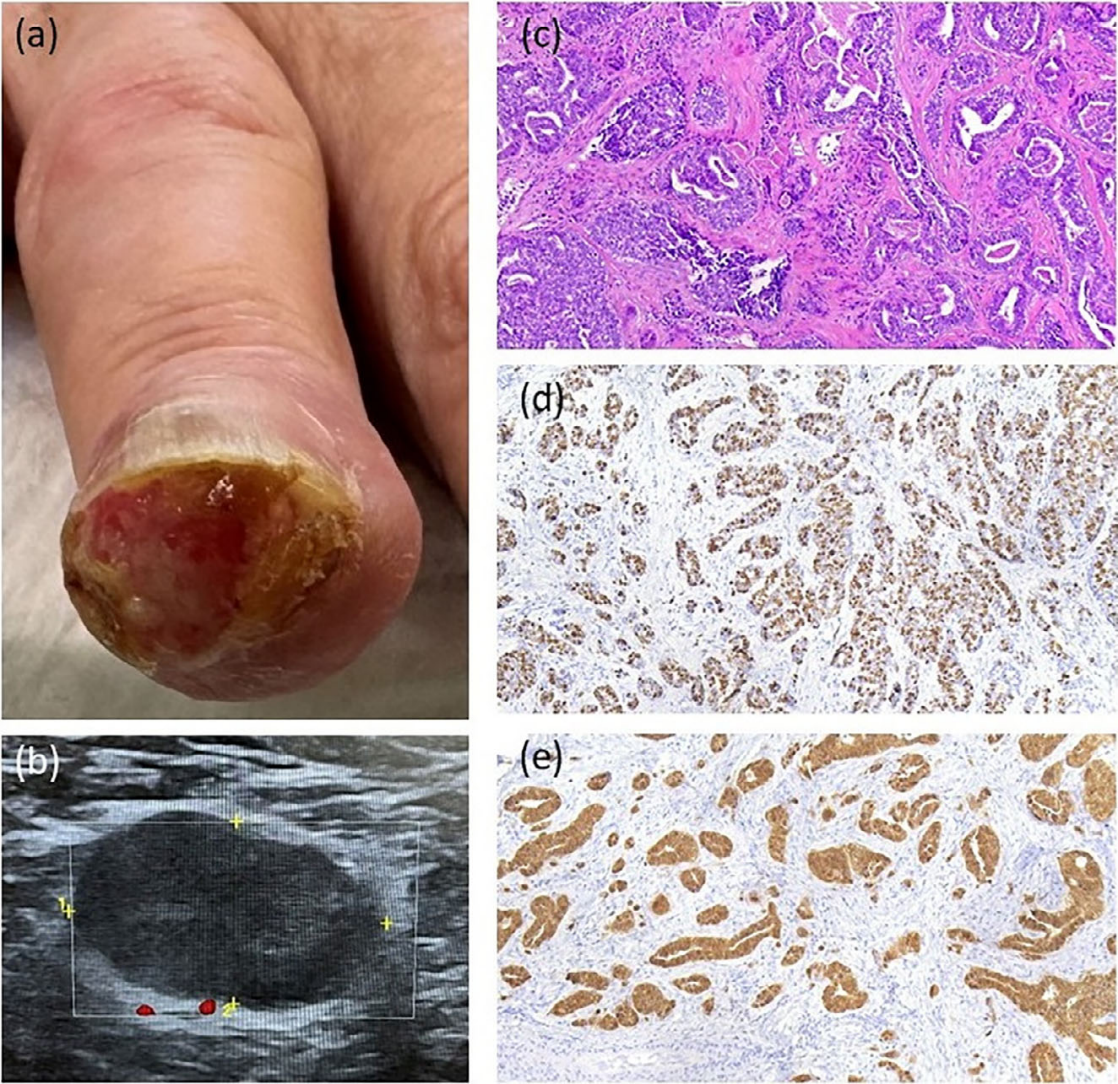
52‐year‐old patient with HPV42‐positive digital papillary adenocarcinoma at the distal phalanx of the left middle finger. (a) Clinical presentation shows an exophytically growing verrucous plaque. (b) Lymph node metastasis: high‐resolution B‐mode ultrasound of the left axilla reveals a pathologically altered lymph node with rounded morphology and a longitudinal‐to‐transverse axis ratio ≤ 2. The internal structure is hypoechoic and heterogeneous. Color Doppler shows peripheral vascular signals but no central hilar blood flow. Histopathology confirmed lymph node metastasis (not shown). (c) Histopathology of the primary tumor shows glandularly organized tumor cells with predominantly basophilic populations and fibrotic stroma (hematoxylin and eosin stain, original magnification × 100). (d) Ki‐67 immunostaining demonstrates a markedly increased proliferation rate in more than 80% of tumor cells (Ki‐67, × 100). (e) p16INK4a staining shows continuous nuclear and cytoplasmic expression of the tumor cells (p16INK4a, × 100).

## DISCUSSION

This case series illustrates that infections with HPV42, currently (still) classified as a LR‐HPV type, can induce not only benign AGW but also squamous cell carcinomas or their precursors on the fingers, as well as DPA, a very rare and aggressive tumor. Overall, HPV42 is a rare HPV type, detected worldwide in only 0.6% of samples from women with normal cytology. Its prevalence among healthy women varies significantly by geography, with the highest prevalence of 1.2% in Africa and only 0.3% in Asia (HPV Information Centre; https://hpvcentre.net/datastatistics.php). In AGW, HPV42 has been detected in 1%–2% of cases.^6^, ^7^ In the presented case of a man with HIV infection (Patient 1), HPV42 was detected as a monoinfection in perianal AGW. It has long been known that LR‐HPVs can rarely induce anogenital carcinomas. In a study including cervical carcinomas from nine countries, LR‐HPV types were found as causative agents in less than 1% of 1739 analyzed tumors, and HPV42 was not detected in any case.^1^ In another study involving 13,328 HPV‐induced carcinomas, HPV42 was detected in only four cases (3 cervical carcinomas and 1 anal carcinoma).^8^ Our research group demonstrated that HPV42 can induce both benign AGW and high‐grade anal dysplasias within a short period of time in the same anatomical area in an immunocompetent patient.^12^

An association of HPV42 with a rare and unusual cervicovaginal lesion, the “seborrheic keratosis‐like lesion” (SKLL), has been described in the literature.^13^, ^14^, ^15^, ^16^ Morphologically, SKLL differs from other intraepithelial lesions by its resemblance to cutaneous seborrheic keratoses (acanthosis and bland, partly basaloid cells with squamoid areas – so‐called “squamous eddies” – and interdigitating hyperpapillomatosis) and the absence of HPV‐induced cytopathic features such as koilocytotic morphology.^16^ In the largest case series analyzing HPV in SKLL to date, HPV42 was detected in 13 of 17 (76.4%) cases, including ten HPV42 monoinfections and three co‐infections (HPV6, HPV16, and HPV61). Two of the investigated SKLLs also showed high‐grade dysplasias with characteristic block staining for p16INK4a.^16^

Unlike UV‐induced epithelial tumors on the dorsum of the hands and finger extensor surfaces, sub‐ and periungual SCC and SCC in situ of the fingers are frequently caused by alpha HR‐HPV types.^17^ In the here described case (Patient 2), HPV42 was detected in digital SCC in situ, and immunohistochemical staining for p16INK4a revealed a “block staining,” strongly suggesting a causal relationship. To our knowledge, this is the first description of a HPV42‐induced digital SCC in situ in the literature. Previous studies by our group on 25 finger SCC and SCC in situ cases, as well as the largest literature review of over 100 HPV‐induced ungual/periungual SCC and SCC in situ (majority caused by HPV16), did not detect HPV42 in any case.^17^, ^18^

DPA is a highly aggressive adnexal tumor originating from sweat glands, almost exclusively localized on fingers and toes.^19^ The incidence of DPA is estimated at 0.08 per 100,000, predominantly affecting men over 50 years of age. Local recurrences after micrographically controlled excision are common, as are lymphogenic and pulmonary metastases.^20^ The presented case (Patient 3) developed lymph node metastasis despite R0 resection of the DPA but has remained recurrence‐free for over 4 years following lymphadenectomy and adjuvant radiotherapy of the affected axilla. The detection of HPV42 by in situ hybridization in primary and metastatic DPA (all 8 investigated DPA cases were HPV42‐positive, with signals exclusively present in tumor cells) was first reported in 2022 and later confirmed in a larger cohort (45 of 47 [96%] of investigated DPA were HPV42‐positive).^9^, ^21^ Expression of the HPV oncogenes E6 and E7 was also demonstrated in DPA tumor cells. HPV42 was detected in five predominantly genitally localized, extremely rare non‐acral DPA as well (5 of 5 cases [100%] HPV42‐positive).^22^ Bayraktar et al.^23^ analyzed 14 DPA cases by targeted DNA/RNA sequencing and gene expression profiling, comparing them with ten hidradenoma cases. Two DPA cases exhibited fusion genes (CRTC3::MAML2 and TRPS1::PLAG1) without a uniform mutation pattern but showed increased expression of genes related to matrix remodeling, metabolism, DNA repair, and activation of the E2F signaling pathway. All tested DPA cases were HPV42‐positive, and immunohistochemistry confirmed elevated expression of CD56, CDC20, SOX10, as well as infiltration by CD20‐positive B cells.^23^ Accordingly, detection of HPV42 may also aid in differentiating DPA from morphologically similar benign sweat gland tumors such as hidradenoma or tubular adenoma.^21^, ^24^, ^25^

In summary, HPV42 can induce benign lesions (e.g., AGW and the rare cervicovaginal SKLL) or malignant lesions (anogenital and digital carcinomas as well as highly aggressive DPA) depending on the infected cell population. The previously classified LR‐HPV type HPV42 can thus be described as a (potentially) oncogenic virus.^25^ The HPV42 E7 oncoprotein can interact with the cellular tumor suppressor protein pRB similarly to the E7 proteins of the established HR‐HPV types HPV16 and HPV18, as evidenced by strong p16 staining in (pre)malignant HPV42‐induced tumors.^8^, ^9^ Likewise, HPV42 E6 binds the E6AP ubiquitin ligase but – unlike HPV16/18 – does not lead to degradation of the tumor suppressor protein p53.^8^, ^9^ In HPV42‐induced DPA, degradation of the tumor suppressor PTPN14 by HPV42 E7 plays a role. The oncogenic potential of HPV42 also appears to depend on the type of infected cells. Unlike anogenital HR‐HPV‐induced carcinomas arising from keratinocytes, DPAs originate from eccrine sweat gland cells, which are abundant on hands and feet.^9^ It remains unclear which additional factors (e.g., environmental factors, ethnicity, patient age) may be necessary to induce malignant transformation by LR‐HPV types such as HPV42.

Currently available nonavalent HPV‐L1‐vaccines prevent over 90% of all HPV‐induced tumors and anogenital warts but do not protect against HPV42‐induced lesions. Even after successful elimination of cervical cancer and other HPV‐associated lesions in countries with high HPV vaccination rates, HPV42 should be considered a causative agent of AGW and rare digital and cervical dysplasias and carcinomas, as it is not covered by currently available HPV vaccine. Future prevention of HPV42‐induced tumors may be possible with emerging pan‐HPV‐L2‐vaccines currently under development.^26^, ^27^

## CONFLICT OF INTEREST STATEMENT

None.
